# Sub-clinical Cervical Root Resorption: A Case Report

**DOI:** 10.7759/cureus.27334

**Published:** 2022-07-27

**Authors:** Ruba F A. Ghazi, Fahad A Khalifa

**Affiliations:** 1 Restorative Department, Specialized Dental Center, King Fahad Armed Forces Hospital, Jeddah, SAU

**Keywords:** incidental resorption, pathologic resorption, cervical resorption, subclinical root resorption, external root resorption, root resorption

## Abstract

External cervical resorption (ECR) is a dynamic pathological process characterized by its cervical position on the root and arises below the epithelial attachment and the coronal part of the bone. This report will highlight a case of ECR in an asymptomatic patient. A radiolucent area was noted during a routine dental follow-up examination on the bitewings at the mesial surface of the upper right second premolar. Persistently, the radiolucency had multiple radiographic views indicating a true form of a lesion that could be diagnosed as external cervical resorption. The patient did not report any complaints since dental treatment was completed in 2016. After a series of radiographs and conducting further diagnostic measures, a diagnosis of ECR was confirmed. The best treatment of choice for this case was extraction and restoring the missing tooth with an implant-supported crown. The size and the extent of such a defect could affect the strategy for implant placement. This paper aimed to report that ECR can be asymptomatic for a long time with advanced cervical root resorption in some cases.

## Introduction

Dental resorption refers to the loss of the tooth structure due to an inflammatory process. The process causes loss of enamel, dentin, and cementum as a consequence of odontoclastic action. It is known to be a clinical challenge due to its complexity [1-2]. Usually, root resorption is desirable in primary dentition, leading to deciduous teeth shedding [3-4]. However, it is also considered a pathological condition when affecting permanent teeth because of its irreversibility. Consequently, it may lead to perpetual injury with imperative dental intervention. In some cases, the affected tooth is extracted and followed by proper esthetic, functional, and biological restoration [4]. Typically, the mineralized tissues of the permanent teeth are not resorbed. Odontoblasts and cementoblasts offer protection to dental hard tissues that form predenin and precementum, respectively. In case of damage, inflammatory multinucleated cells will colonize the mineralized or denuded surfaces, initiating resorption [5].

Dental resorption is classified based on the affected root surface as either internal resorption or external resorption [4]. External resorption is a form of root resorption that originates on the root’s exterior surface, which may invade dentin in any direction and to a varying degree [2]. It can also be further divided into surface resorption, external inflammatory resorption, external replacement resorption, transient apical resorption, and external cervical resorption (ECR) [4]. ECR is an uncommon and aggressive form of external resorption. It is defined as a dynamic pathological process characterized by its cervical position on the root. It arises below the epithelial attachment and the coronal part of the bone, known as the connective tissue attachment. It progresses into different parts of the periodontium circumferentially and apico-coronally. Forming resorptive channels inside the root may terminate at the pulpal tissue and cause substantial damage to the tooth [3].

The same phenomenon of aggressive cervical root resorption is also recognized by different terms, such as invasive cervical resorption, odontoclastoma, idiopathic external resorption, fibrous dysplasia of the teeth, burrowing resorption, late cervical resorption, extra-canal invasive resorption, peripheral cervical resorption, supra-osseous inflammatory root resorption, subepithelial inflammatory resorption, and periodontal infection resorption [1]. The ECR that has been studied in previous literature was reported with a prevalence rate ranging from 0.02 % to 0.08% [3].

Heithersay studied the extent of the ECR and came up with what is known later as Heithersay’s classification [6]. The first classification in 1999 used periapical radiographs, and he divided them into four classes depending on the size and the extent of the resorption process in dentin. The classifications are Class I; a small lesion, which extends up to the cervical area of the tooth and a slight penetration into the dentin. Class II; small, but well-defined lesion with deeper penetration of the defect towards the pulp chamber, with a little or no involvement of the root circumferentially. Class III; a large lesion that extends to the root dentin of the coronal third. Class IV; a large and invasive lesion in which the defect extends beyond the coronal third of the root.

Many studies have investigated the different potential etiologies for ECR. They concluded that there is a field of uncertainty to point out one reason to be the trigger for initiating the resorption. Instead, there are potential predisposing factors [3]. The importance in determining the possible etiologies and discovering them in the early stages is to limit the detrimental effect of the disease [7]. Heithersay was interested to study the predisposing factors extensively among 257 cases of ECR in 222 patients. He found that the most common factors were orthodontic treatment, dental trauma, oral surgery, periodontal therapy, bruxism, intracoronal restoration, delayed eruption, enamel stripping, dento-alveolar defects, and poor oral hygiene and restoration [2-4]. Orthodontic treatment has accounted for 45% of the cases, while dental trauma accounted for 28.5%. Meanwhile, the maxillary central incisors are the most affected tooth (29%) [8]. The result of the study indicated that ECR is multifactorial in nature [8].

Clinically, it can vary from being asymptomatic in the early stages when no pulpal involvement is detected; at this point it will be diagnosed more likely during routine dental exam. Or it may appear as a pink or red discoloration that may develop at the cervical part of the tooth. If it appears as a discoloration it will be difficult to diagnose especially if it is located in the posterior teeth. When this occurs, that may alert the patient.

This discoloration is the action of granulation tissue in the dental tissue; if the granulation tissue extends and perforates enamel or dentin at the gingival margin only then it may cause mild gingival hyperplasia and may bleed then it will become symptomatic, which was not reported in our case, although the lesion started for few years undetected and involved up to the mid-root surface area [2]. In the highlighted case below, symptoms were completely sub-clinical although the extent of the resorption was advanced, but no detection of any periodontal symptoms including swelling, gingival hyperplasia, or bleeding [2].

Early stages of ECR lack apparent symptoms, which poses difficulty for clinicians to identify and diagnose the condition. Diagnosis is made based on the radiographic findings. The discovery of cone-beam computed tomography (CBCT) significantly improved the assessment and detection of the lesion with high reliability and effectiveness [9]. A preoperative CBCT can help clinicians create an effective treatment plan by accurately assessing the resorption and eventually conducting the proper treatment strategy, which is exactly how it provided detection of the defect in depth for the reported case. A conventional radiograph may not aid in identifying the condition especially when the lesion is minimal or due to its location that may be extending apical or coronal from the cervical lesion [2]. Therefore CBCT had an effective role in the highlighted case to identify the extent and furthermore in proper assessment of the lesion.

The presented case below was entirely identified during a routine dental examination. The patient was not complaining of any signs or symptoms. The progress of the condition started in 2019. This paper aimed to report that ECR can be asymptomatic for a long time in some cases with advanced classification of cervical root resorption.

## Case presentation

A 35-year-old healthy female patient presented to the dental clinic for a routine checkup. After taking bilateral bitewing radiographs, a band of radiolucent area was evident on the mesial surface of the second premolar.

**Figure 1:**
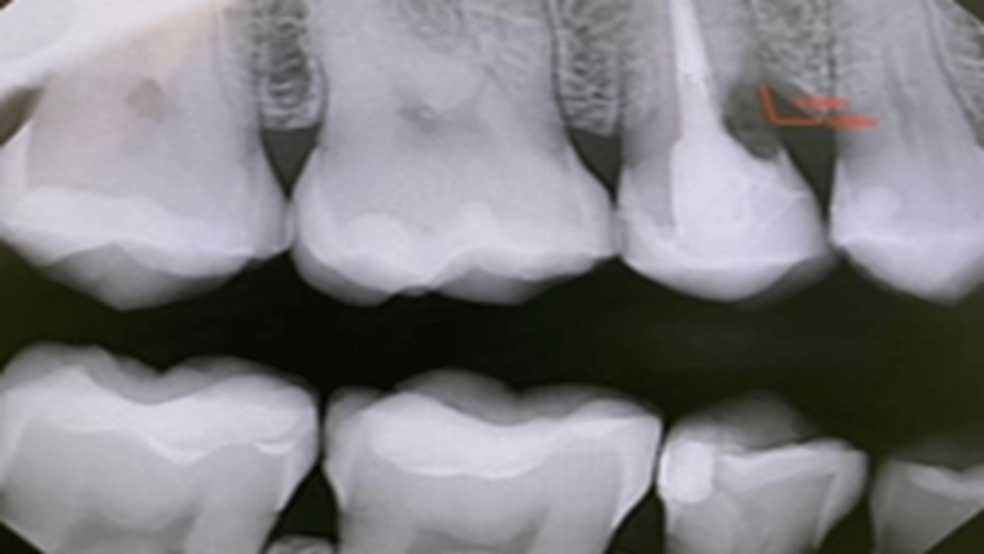
Mesial radiolucent area for approximating the crestal bone.

Radiograph was repeated with multiple angles to confirm the finding and trace the lesion in that area; the lesion appeared mesial to tooth #15 with evident vertical bone loss extending beyond the cemento-enamel junction (CEJ) (Figure 1). 

**Figure 2:**
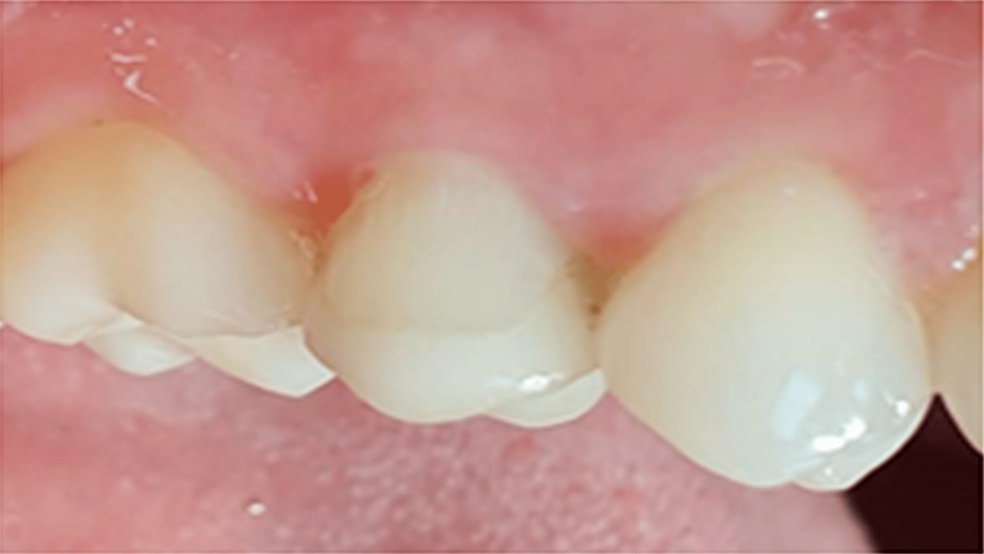
At the time of routine dental exam and without report of any significant signs or symptoms.

Clinically, the tooth had a ceramic onlay, which was done in 2016, with unremarkable significant symptoms (Figure 2). The only significant clinical finding was a deep pocket on the mesial aspect of the tooth that measured around 5-6 mm. Percussion and palpation were within normal range, and there were no findings or reports of bleeding, gingival hyperplasia or previous swelling.

**Figure 3:**
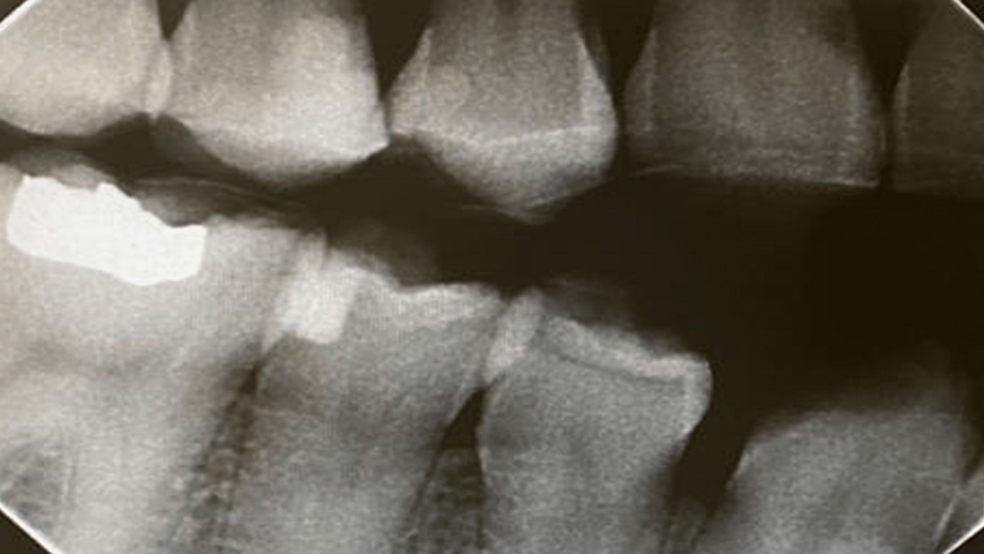
Bitewing radiograph showing the tooth 15 with mesial composite restoration approximating to pulp horn.

**Figure 4:**
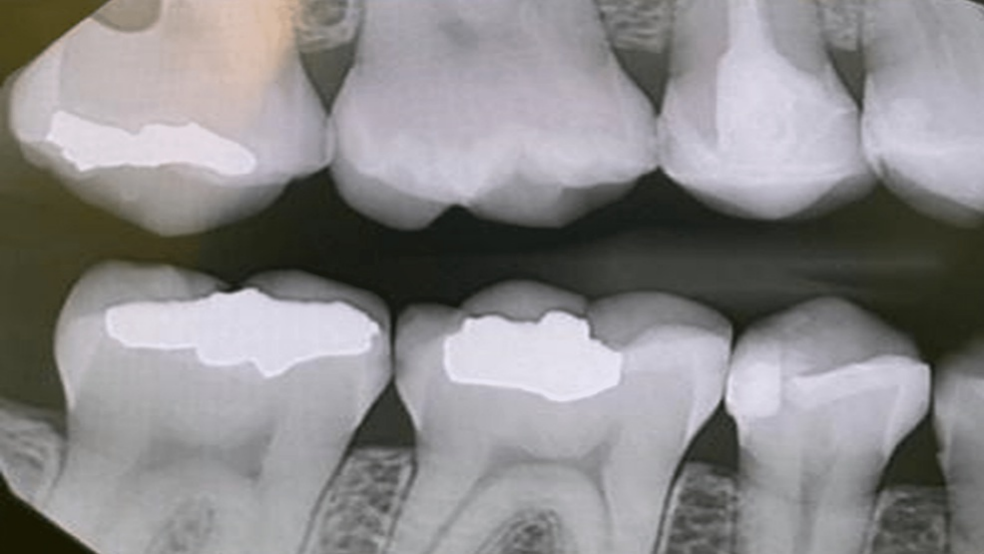
After root canal completion and cementing the indirect ceramic restoration.

The dental history of tooth #15 started with a deep composite restoration in 2014 (Figure 3). This later led to pulpal involvement after persistent pain that required root canal treatment, which was later followed by an indirect ceramic onlay at the end of 2016 (Figure 4).

**Figure 5:**
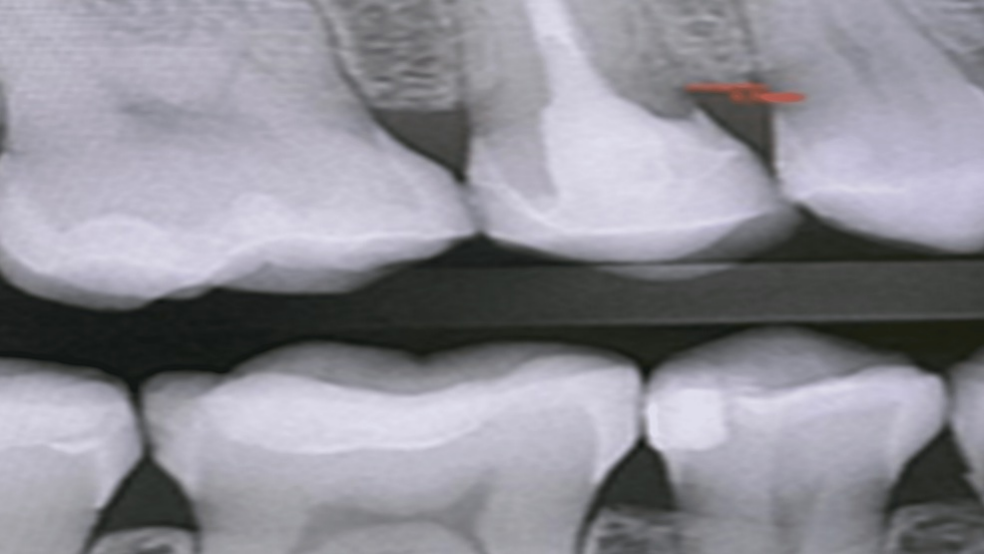
The start of the resorptive activity later diagnosed as external cervical resorption (ECR).

**Figure 6:**
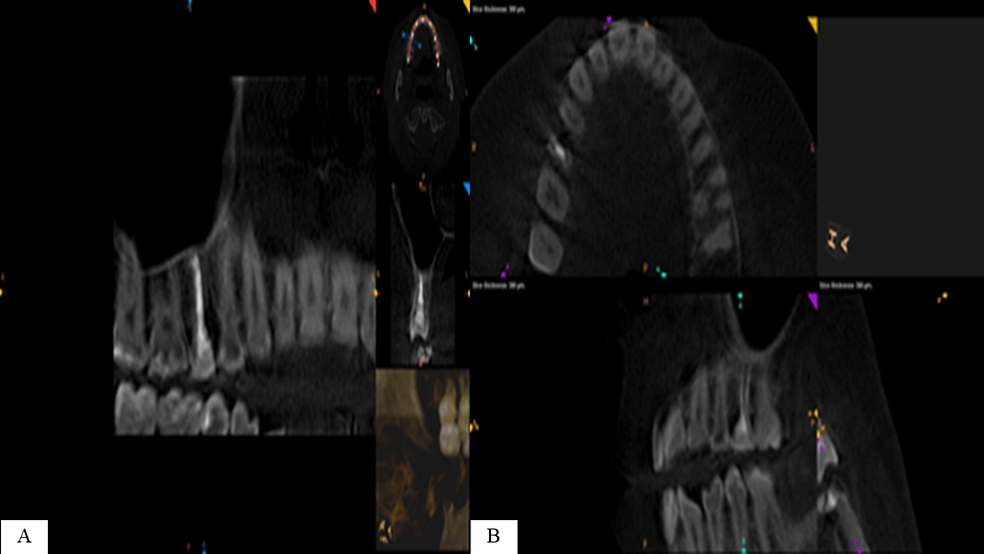
A: Axial view of the cone-beam computed tomography (CBCT) shows area of discontinuity of alveolar bone on the mesial surface of tooth 15. B: Sagittal view of the CBCT showing tooth 15 with mesial angular defect extending up to the mid-part of the root.

Revising previous dental radiographs of the same side that were recorded during routine maintenance dental visits around 2018 showed that the lesion started to appear but was missed radiographically (Figure 5) and after conducting a series of examinations and utilizing the CBCT, a definitive diagnosis of ECR was confirmed (Figure 6).

The treatment strategy was to maintain the affected tooth in a healthy and functional status on the dental arch and improve esthetics. Specifically, the first treatment option was to open a flap, curette the defective lesion, and explore the neighboring premolar for possible involvement. The second line of treatment was to extract the tooth and replace it with an implant, which was the treatment of choice considering the procedural prognosis, success rate, and esthetics, to replace the affected tooth.

Treatment

**Figure 7:**
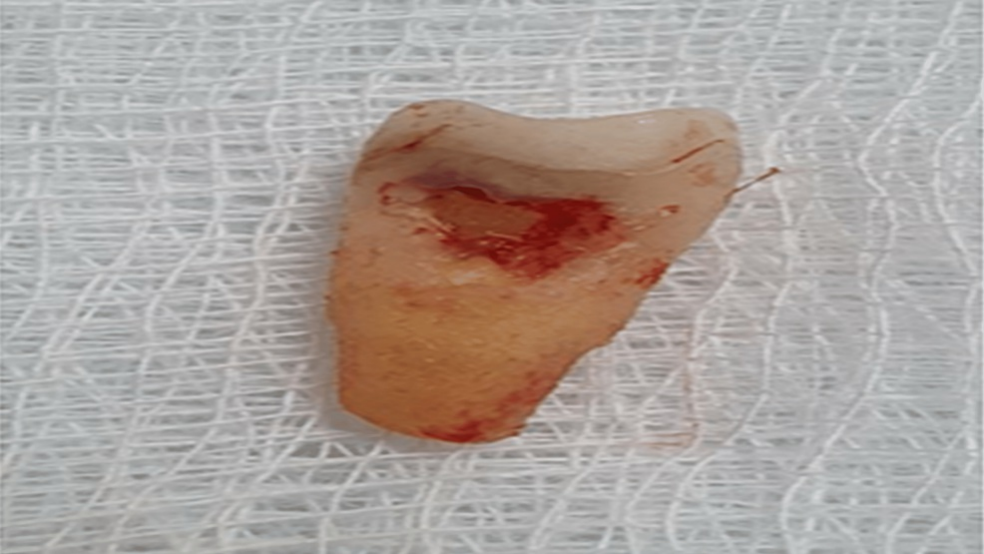
Extracted tooth showing the side view of the resorptive cavity extending at the mesial surface up to the mid-surface of the root.

**Figure 8:**
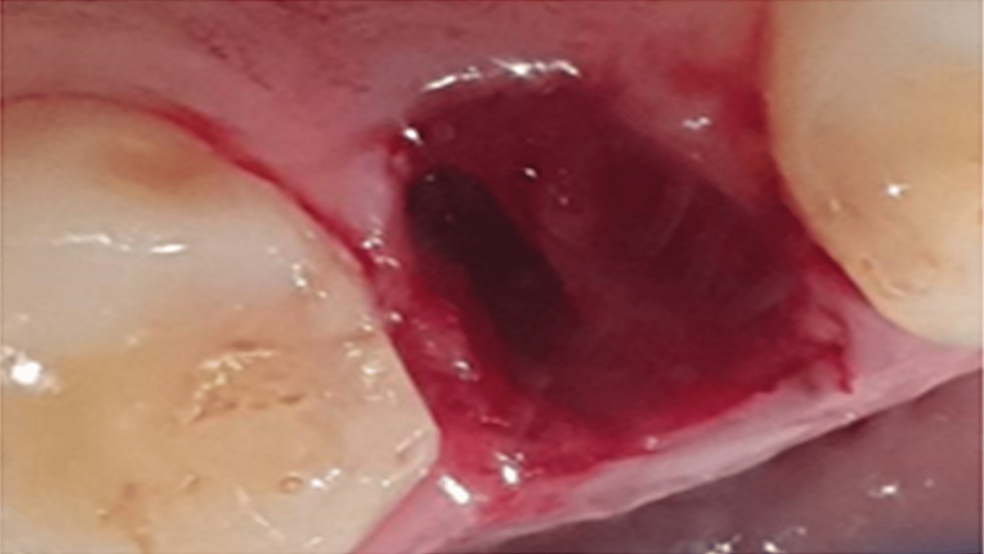
Extraction socket was intact with no signs of discontinuity or resorption.

Pre-operatively, a surgical stent was fabricated with clear autopolymerizing polymethyl-methacrylate resin. Because of the high esthetic demand of the patient, an immediate non-loaded crown was used. Intra-operatively, a block anesthetic technique was chosen to anesthetize the anterior superior alveolar nerve and the palatal nerve. Local anesthesia was administered (scandicaine with 1:100,000 epinephrine), and atraumatic extraction was then performed using the periotome to preserve the buccal plate. The extracted tooth shows that the resorption was evident and extended up to the middle surface of the root (Figure 7). Then the socket was inspected for any defects or signs of bone resorption and possible damage in the neighboring teeth (Figure 8).

**Figure 9:**
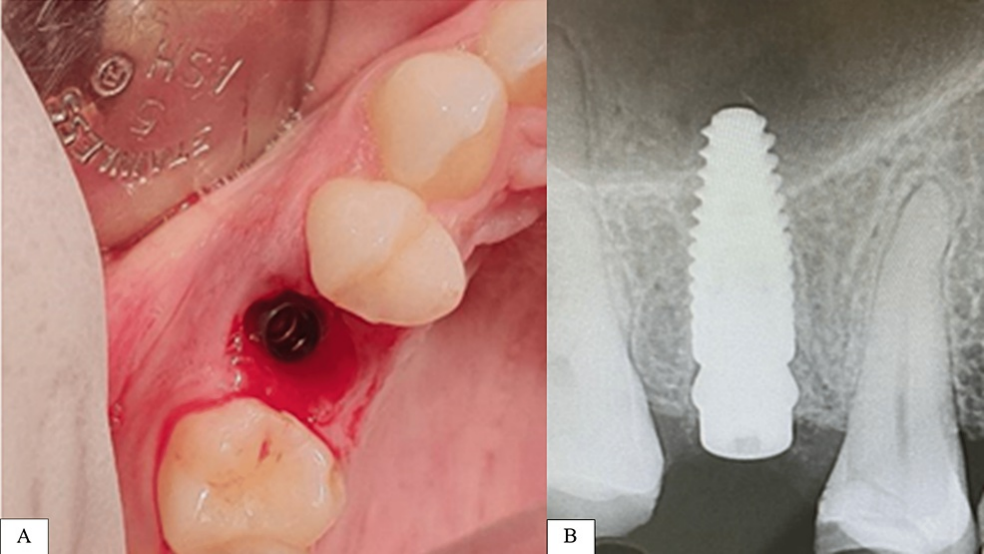
A: Clinical view for the implant insertion site. B: Radiographic view for the implant.

**Figure 10:**
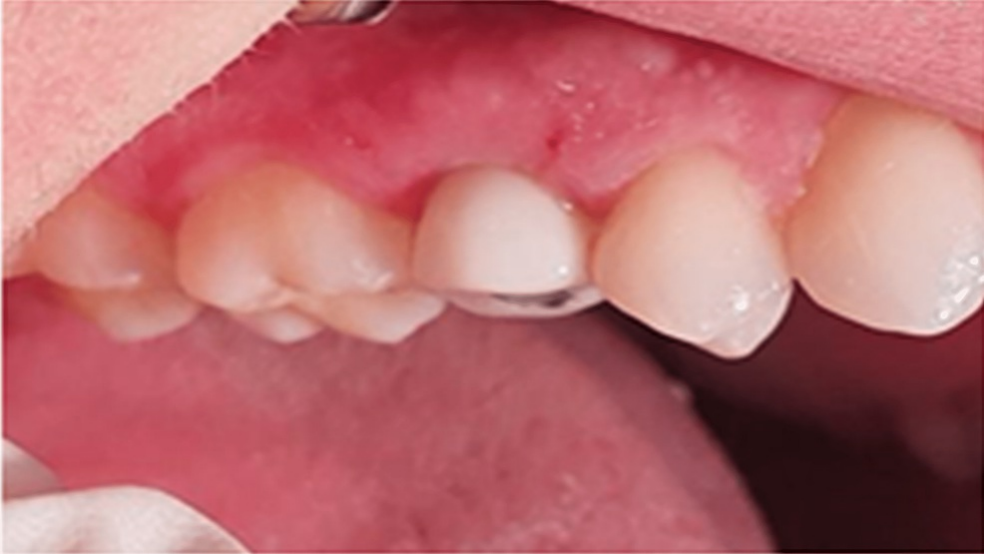
Non-functional loading of the provisional crown.

Proceeding with the immediate implant insertion (Straumann, Basel, Switzerland; bone level 4.1 × 10) bone-level tapered neck as the clinical and periapical radiograph shows in Figure 9. Attention was carried to engaging the implant into the palatal wall of the extraction socket to obtain stability. The temporary crown was loaded in a non-functional form within 72 hours after implant insertion (Figure 10). Post-operative instructions were provided to the patient, including 0.2% chlorhexidine mouth wash twice daily for seven days.

**Figure 11:**
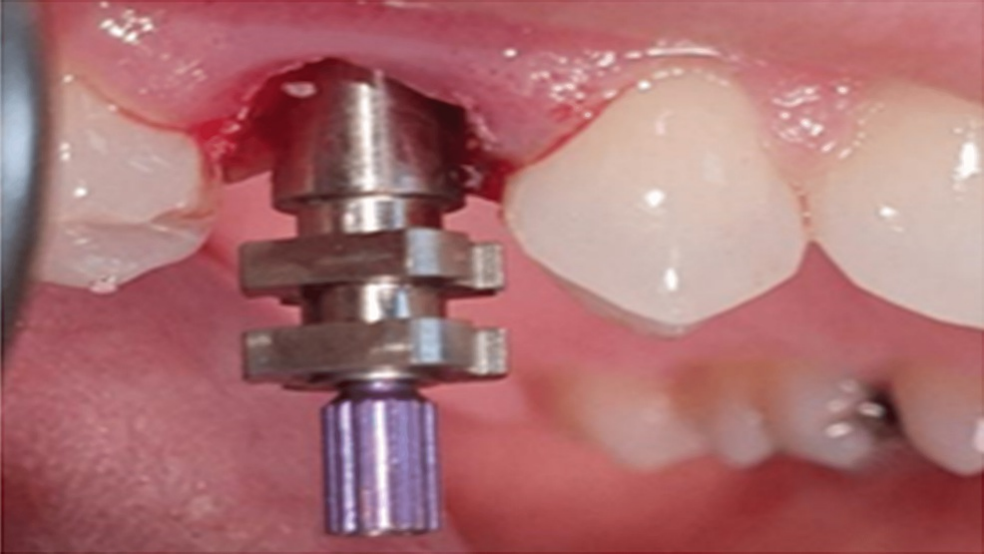
Impression post for open-tray impression technique.

**Figure 12:**
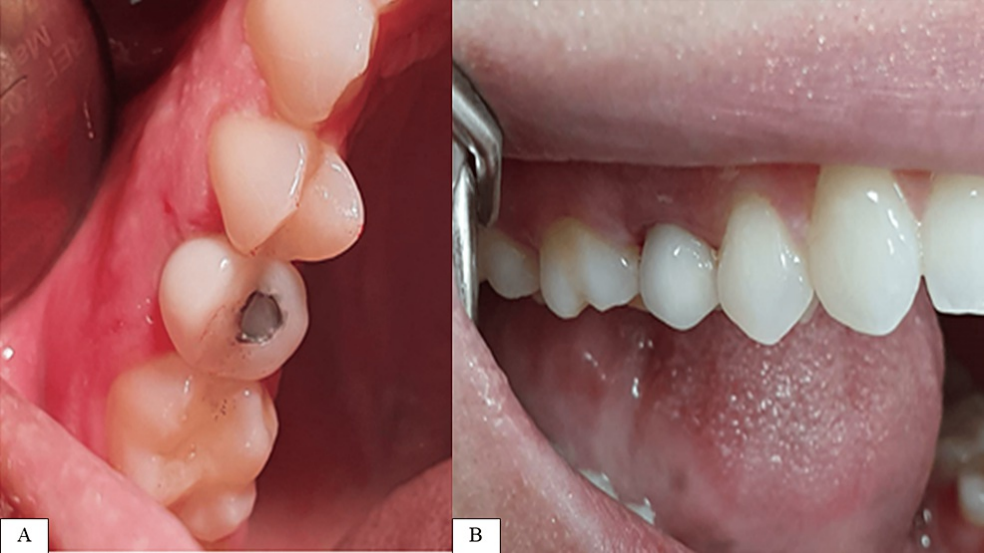
Post insertion of screw-retained all-ceramic crown. A: Occlusal view. B: Lateral view.

**Figure 13:**
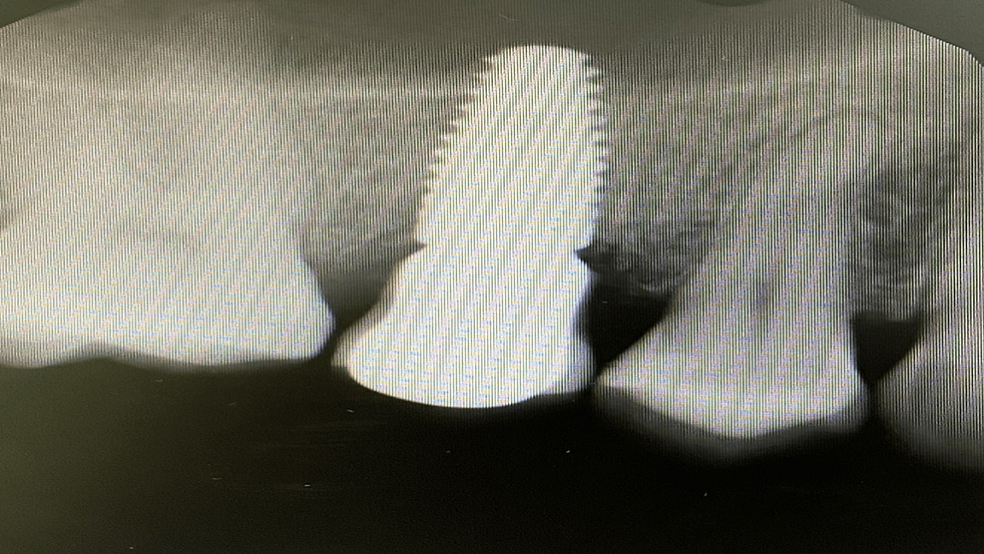
A periapical x-ray showing two-year follow-up status.

Three months later, the final impression was completed using the open tray impression technique with polyether impression material (Figure 11). Insertion of digitally fabricated B2-shade zircon crown took place (Figure 12). The first follow-up visit was within three months, evaluating the occlusion, stability, or any signs of instability. The second visit was at the end of six months, then at 12 months, and eventually two-year follow-up (Figure 13).

## Discussion

Literature has agreed that, due to the broad range of predisposing factors for this disease [7], there is no classic presentation of ECR, and clinical and radiographic examinations discover many cases incidentally [10]. Some cases of ECR arise in patients without any predisposing factors. They are found in completely healthy individuals, with the only significant finding being hypothyroidism [11]. This case is reported in a healthy individual, with a history of root canal treatment that was performed in 2016. Since then, no signs or symptoms have been reported during a routine dental examination although based on Heithersay's classification for ECR, our presented case above is considered as class III which is characterized by a large lesion that extends to the root dentin of the coronal third.

ECRs are lesions known for their aggressive nature, and they resist intervention, which may eventually end in tooth loss [12]. As explained previously, ECR is asymptomatic in the early stages. The patient may later present with symptoms once the pulp becomes involved [10]. Clinical signs and symptoms may be the first indication of the arising problem, including discoloration, spontaneous localized pain, tenderness on percussion, palpation, or mastication. Sometimes, it may involve a draining sinus or a buccal swelling [2]. When the lesion occurs on the buccal or lingual surfaces, the diagnosis can be easily made during a routine examination, and sometimes it may be mistaken for cervical caries [10].

It is crucial to base the diagnosis of ECR on a proper diagnostic tool and skills, such as tactile sense during examination [10]. Moreover, the utilization of a reliable radiographic technique such as the CBCT whenever possible was reported by Patel et al. in 2009, when they noted a significant prevalence (p=0.028) for proper diagnosis CBCT compared with a conventional radiograph [9].

Management of this case took place during routine dental examination, which is atypical to most cases where symptoms have to develop at some point. Although no expected pulpal symptoms will develop since the tooth is endodontically treated in the first place; yet no periodontal symptoms were noted that explain the extent of the defect on the root and the degree of resorption. This case is similar to a reported case series written by Murugadoss et al. in 2021 that was utterly iatrogenic in nature [13]. It is necessary to highlight the importance of a multidisciplinary approach, from a careful medical examination to a thorough dental evaluation, to contain the resorptive lesion’s aggressive nature and provide the best dental intervention [12].

## Conclusions

Several potential predisposing factors have been identified for ECR. However, more research is required to investigate their causality, as ECR is more prevalent with certain combinations of these predisposing factors. Also, encouraging the use of the CBCT as the conventional radiograph will view the defect in two-dimensional aspects, especially if the lesion approximates more toward the buccal or labial surface. Otherwise, the ECR might progressively occur asymptomatically and be discovered only during a routine examination.
